# The Impact of Mind-Body Medicine on Patient-Reported Outcomes in the Management of Chronic Pelvic Pain

**DOI:** 10.7759/cureus.62376

**Published:** 2024-06-14

**Authors:** Aakriti R Carrubba, Kristin A Lothman, Colleen S Ball, Amy L Mongan, Adam I Perlman, Anita Chen

**Affiliations:** 1 Medical and Surgical Gynecology, Mayo Clinic, Jacksonville, USA; 2 Integrative Medicine, Mayo Clinic, Jacksonville, USA; 3 Statistics, Mayo Clinic, Jacksonville, USA; 4 Nursing, Mayo Clinic, Jacksonville, USA; 5 Integrative Medicine, Pendulum Therapeutics, San Francisco, USA

**Keywords:** mindfulness, integrative medicine, patient-reported outcomes, chronic pelvic pain, mind-body counseling

## Abstract

Background

Recent research has suggested a role for mindfulness-based therapy for patients with chronic medical conditions, but there is limited data on pelvic pain. We aim to determine if mindfulness improves patient-reported outcomes in pelvic pain and to determine the feasibility of implementation of this program.

Methodology

This is a pilot feasibility trial consisting of women with chronic pelvic pain at a single academic tertiary referral clinic. A convenience sample of 15 subjects was enrolled. Subjects were scheduled for three 60-minute virtual mind-body sessions with a certified counselor. Baseline scores were obtained using the Patient-Reported Outcomes Measurement Information System-Computer Adaptive Testing (PROMIS-CAT) platform. They were repeated three months and six months after enrollment. Descriptive statistics were performed.

Results

A total of 15 patients were enrolled in the study. Among the 13 patients who completed the three-month PROMIS-CAT scores, seven had a clinically significant 5-point improvement in sleep disturbance T-score. At least a 5-point improvement in fatigue, pain interference, and ability to participate in social roles and activities T-scores were observed in six patients each. There was a 40% dropout rate.

Conclusions

A formal mind-body counseling program can support patients with chronic pelvic pain. Our trial demonstrated the feasibility of establishing a program and modest improvement in patient-reported quality of life.

## Introduction

Patients with chronic illnesses [1] often need to direct and self-advocate for their treatment. Barriers to care include limited time during appointments, consultations with different providers, technical communication style, and health literacy. Patients with chronic pain syndromes experience symptoms that can negatively impact their quality of life by interfering with their ability to sleep, work, and function in their social roles. Pelvic pain can also affect sexual function, relationships with partners, fertility desires, reproductive function, and bowel or bladder function. There are many stigmas associated with chronic pelvic pain which lead to further disconnect and isolation.

One potential therapeutic intervention for chronic pain management is mindfulness. A concept under the larger category of mind-body medicine, mindfulness is the intentional and non-judgmental conscious awareness of the present moment, or “paying attention on purpose” [2,3], with the goal of physical and psychological health improvement. Participants are taught autonomy, increasing self-regulation, and focusing awareness on a particular goal or activity [3]. Mindfulness-based stress reduction in chronic pelvic pain has been studied with mixed findings [2]. A limited quality systematic review [4] suggested that mindfulness training versus waitlist control significantly improved pain acceptance and depression scores for patients with low back pain, fibromyalgia, tension headache, and general chronic pain, but did not significantly improve pain intensity, anxiety, and quality of life outcomes. Another systematic review evaluating mindfulness-based therapy in sexual dysfunction [5,6] found improvements in arousal, desire, and sexual satisfaction; a reduction of fear linked with sexual activity; and improved consistency between arousal and genital response in women.

A randomized controlled trial [7] utilizing a smartphone application compared patients with chronic pelvic pain in a 1:1:1 ratio of active interventions consisting of structured mindfulness meditation, active controls with non-meditative muscle relaxing instructions, and a “treatment-as-usual” group. There were high recruitment and follow-up rates [8], but low adherence (only one to seven days of mean app use over 60 days). There were no differences in pain acceptance scores among the groups. In contrast, weekly mindfulness-based cognitive-behavioral therapy in patients with provoked vestibulodynia was superior to traditional cognitive-behavioral therapy over an eight-week group in-person treatment course in pain intensity with intercourse/penetration [9]. Both groups had a reduction in sexual dysfunction, sexual distress, pain catastrophizing, and pain hypervigilance, along with increased pain acceptance; effects were maintained at the six-month follow-up. These studies highlight the fact that technology-based treatments have limited patient engagement and require highly motivated users. Another recent trial [10] demonstrated that psychoeducational cognitive-behavioral counseling can improve female sexual function, evidenced by improvement in Female Sexual Function Index scores after eight counseling sessions in patients randomized to the intervention.

We developed a mind-body medicine program specifically for women with pelvic pain which would serve as an adjunct to their current medical or interventional therapy. We believe that a formal treatment program consisting of both individual and group sessions with a mind-body counselor will improve patient engagement and promote adherence to pain-coping therapy. The primary outcome of our study is to determine if there is an improvement in patient-reported quality of life outcomes in women undergoing treatment for chronic pelvic pain. Secondary outcomes are to assess feasibility, waiting times, and appointment attendance in patients receiving mind-body services.

## Materials and methods

This is a pilot feasibility trial consisting of a convenience sample of women with a diagnosis of chronic pelvic pain who are being seen for gynecologic care at a single academic tertiary referral clinic. The study was proposed and accepted for funding through an institutional grant called the Research Accelerator for Clinicians Engaged in Research (RACER) program. The study was approved by the Mayo Clinic Institutional Review Board and marked exempt from continuing review as there was no greater than minimal risk to participants (IRB# 21-000221). The study was registered on the clinicaltrials.gov website on February 23, 2021 (ID number: NCT04773925). Inclusion criteria were English-speaking adult women aged 18 through 89 with a diagnosis of chronic pelvic pain (non-cyclical abdominal and pelvic discomfort lasting for greater than six months) who were able to provide consent for participation. Subjects were required to have access to the electronic medical records portal to participate in the program. Recruitment occurred during gynecology outpatient appointments, at which time patients were given documents explaining the study. Written informed consent was obtained and retained by the research coordinator. Exclusion criteria were patients with diagnoses of non-gynecologic pain, acute pelvic pain lasting less than six months, and active abdominal or pelvic malignancy. The goal enrollment was 15 subjects based on available funding and logistics.

After enrollment, each patient was scheduled for three, 60-minute mind-body counseling sessions with a Licensed Clinical Social Worker, who also maintained additional certifications including but not limited to a national certification for mind-body medicine, health and wellness coaching, and a 200-hour yoga certification. Due to COVID-19 precautions and physical distancing restrictions, all sessions were offered virtually. In the initial session, each patient met with the mind-body medicine counselor individually to accomplish the following: (1) to establish trust and safety between the patient and provider; (2) to deeply listen to and provide a compassionate understanding of the patient’s pain experience, while maintaining a biopsychosocial and trauma-informed perspective; (3) to explore what holistic recovery and stress resilience looks and feels like to the patient; (4) to ask thoughtful questions that evoke awareness around the patient’s core values and beliefs around pelvic pain and what might be getting in the way of her progress; and (5) to clarify the patient’s willingness and commitment to participate in the pilot program. Following the initial session, participants were sent through their patient portal the following information: (1) a program outline and how to prepare for sessions; (2) a Mayo-approved booklet on chronic pelvic pain; (3) Duke Integrative Medicine’s Wheel of Health; and (4) Mind-Body Medicine Counselor’s guiding principles and standards for presence.

Subsequently, patients were placed into groups of up to five participants, and the remaining two sessions consisted of group counseling. During these sessions, patients were encouraged to discuss coping resources and their personal experiences with each other and the counselor. In the initial group session, mindfulness-based group norms were clarified and agreed upon, and then psychoeducation was provided on the healing aspects of mindfulness and how it is foundational to holistic health and stress resilience practice. The mind-body medicine counselor guided the group in a mindfulness meditation closely modeled after Dan Seigel’s Wheel of Awareness [11] practice with additional trauma-sensitive cueing. This was followed by group sharing, support, and action planning. Presentation slides and recommended practices were also sent through the portal. The third and final group session started with a heart-centered breathing meditation, which then transitioned into a group check-in on present moment experience as well as progress on action steps. The counselor then provided instruction on basic breathing practices to help the patient learn various ways to support oneself in compassionate awareness and state shift out of a stress reaction in real-time. Additional self-care practices such as sleep hygiene, progressive muscle relaxation, and gentle yoga were also recommended and additional resources were sent through the portal. Patients were also encouraged to reflect on the program experience, identify key insights, and that they need to take continued action to support their health moving forward. A standard script was not used, and the sessions were tailored based on the needs of the participants while adhering to the themes listed above.

Patient-reported outcome scores were obtained using the Patient-Reported Outcomes Measurement Information System-Computer Adaptive Testing (PROMIS-CAT) platform, funded by the National Institutes of Health. The goal of this platform is to provide efficient, valid, and responsive measures of health and well-being without the excessive burden of long questionnaires. Multiple studies [12,13] have validated the use of PROMIS in a range of clinical conditions, including pain syndromes. In our study, we selected seven PROMIS-CAT domains that are relevant to a chronic pain population, namely, depression, anxiety, pain interference, physical function, fatigue, sleep disturbance, and ability to participate in social roles and activities. PROMIS-CAT score ranges differ [13] based on the variable and have been published elsewhere.

In terms of PROMIS-CAT scoring, prior studies have suggested minimal clinically important difference (MCID) score thresholds to determine the effectiveness of treatment options for a variety of conditions. In cervical spine surgery, for example [14], one study suggested an MCID of 4.5 to 4.9 points for pain interference and physical function categories. For anterior cruciate ligament reconstruction [15], MCID was 4.5 for physical function categories and 5.4 points for pain interference categories. Another study [16] for chronic low back pain suggested a cut-off of 5.0 points as an appropriate MCID for most PROMIS domains. There are no current suggested MCID levels for pelvic pain, so we used a cut-off of 5 points based on these prior studies for our analysis. Questionnaires were assigned to patients using the electronic medical records portal at the time of enrollment (baseline), and they were repeated at three months and six months after enrollment to assess for change.

The electronic medical record of each subject was then reviewed for the clinical course, including pain history and examination findings, medical or surgical interventions during the study period, Emergency Department visits, messages and phone calls, and medical comorbidities. Attendance and waiting times for mind-body visits were recorded. All data was stored in a de-identified RedCap database which was only accessible to the study investigators. Statistical analysis was primarily descriptive. All data underwent analysis with descriptive statistics using an intention-to-treat approach. PROMIS-CAT T-scores at baseline, three months, and six months were graphically displayed using spaghetti plots. All statistical analysis and graphics were created using R version 4.0.3 (R Foundation for Statistical Computing, Vienna, Austria).

## Results

A total of 15 participants met the inclusion criteria and were enrolled in the study. The participants’ age ranged from 22 to 68 years (median = 43 years). Participant demographics are described in Table 1. The most common pain symptoms were pelvic and perineal pain (N = 12), abdominal pain or bloating (N = 11), dyspareunia (N = 11), and bowel symptoms (N = 9). Pain symptoms are described in Table 2.

**Table 1:** Participant demographics (N = 15).

	Median (minimum, maximum) or n (%)
Age at time of enrollment	43 (22, 68)
Race	
White	12 (80%)
Black	1 (7%)
Asian	0 (0%)
Pacific Islander	0 (0%)
Other	2 (13%)
Ethnicity: Hispanic or Latino	0 (0%)
Marital status	
Single	4 (27%)
Married or committed relationship	11 (73%)
Insurance	
Private/Commercial	12 (80%)
Medicare or Medicaid	3 (20%)
Smoking status	
Never smoker	9 (60%)
Former smoker	6 (40%)
Menopausal status	
Pre-menopausal	8 (53%)
Post-menopausal	4 (27%)
Unknown or not reported	3 (20%)
Gravida	1 (0, 6)
Parity	1 (0, 2)
Number of vaginal deliveries	0 (0, 6)
Body mass index	29.9 (18.2, 53.1)

**Table 2:** Pain characteristics (N = 15).

	Median (minimum, maximum) or n (%)
Duration of pelvic pain (months)	30 (6, 108)
Pain symptom(s)	
Pelvic and perineal pain	12 (80%)
Abdominal pain or bloating	11 (73%)
Back pain	2 (13%)
Dysmenorrhea: pain only present during menses	3 (20%)
Dyspareunia	11 (73%)
Urinary symptoms: incontinence, dysuria, or urinary retention	4 (27%)
Bowel symptoms: constipation, dyschezia, or diarrhea	9 (60%)
Other	1 (7%)
Prolapse	0 (0%)

Table 3 describes medical comorbidities and medical history. The most common medical comorbidity was anxiety or depression (N = 10). The most common prior gynecologic surgical treatments included hysterectomy (N = 6), adnexal surgery (N = 7), and diagnostic laparoscopy for a gynecologic indication (N = 7). The most common imaging finding was evidence suggestive of deep infiltrating endometriosis (N = 5). The most common treatments for pelvic pain included hormones (N = 8), pelvic floor physical therapy (N = 8), non-steroidal anti-inflammatory drugs (N = 6), and antidepressants (N = 5). Most participants had co-management with the Gastroenterology service for their pain (N = 11). The most common pelvic examination findings included trigger points and tenderness in levator ani muscles (N = 12) and trigger points and tenderness in obturator internus muscles (N = 12).

**Table 3:** Medical comorbidities and medical history (N = 15).

	n (%)
Medical comorbidities	
Gastrointestinal disorders	6 (40%)
Endometriosis	6 (40%)
Chronic pain syndromes	6 (40%)
Urinary disorders	0 (0%)
Vulvovaginal disorders	0 (0%)
Diabetes	2 (13%)
Cardiovascular disorders	4 (27%)
Thyroid disorders	2 (13%)
Cancer	3 (20%)
Anxiety or depression	10 (67%)
Other	4 (27%)
Prior gynecologic surgical treatment(s)	
Hysterectomy	6 (40%)
Pelvic reconstruction surgery	0 (0%)
Anti-incontinence procedure	1 (7%)
Adnexal surgery	7 (47%)
Diagnostic laparoscopy for gynecologic indication	7 (47%)
Bowel procedure	1 (7%)
Cesarean section	2 (13%)
Other gynecologic procedure	1 (7%)
None	4 (27%)
Imaging done before enrollment in the study for workup of pelvic pain	
Ultrasound	3 (20%)
CT scan	4 (27%)
MRI	10 (67%)
None	2 (13%)
Imaging findings	
Uterine fibroids	1 (7%)
Adenomyosis	1 (7%)
Endometrial polyps or thickened endometrial stripe	0 (0%)
Abnormal sliding sign (ultrasound)	0 (0%)
Ovarian cysts	2 (13%)
Evidence suggestive of deep infiltrating endometriosis	5 (33%)
Hydronephrosis	0 (0%)
Bowel endometriosis	0 (0%)
Other	2 (13%)
Prior treatments for pelvic pain	
Benzodiazepines (oral)	0 (0%)
Antidepressants	5 (33%)
Opioids	3 (20%)
Non-steroidal anti-inflammatory drugs	6 (40%)
Muscle relaxants (oral)	3 (20%)
Vaginal suppositories (muscle relaxers)	2 (13%)
Neuroleptic medications	1 (7%)
Cannabinoids	3 (20%)
Trigger point injections	0 (0%)
Botox injections	2 (13%)
Pelvic floor physical therapy	8 (53%)
Acupuncture	1 (7%)
Yoga	1 (7%)
Cognitive behavioral therapy	0 (0%)
Pain rehabilitation clinic	1 (7%)
Other	4 (27%)
None	1 (7%)
Hormone therapy	8 (53%)
Gonadotrophic-releasing hormone agonists or antagonists	1 (7%)
Co-management with other sub-specialty services	
Gastroenterology	11 (73%)
Physical Medicine and Rehabilitation	4 (27%)
Rheumatology	2 (13%)
Pain Rehabilitation Center	2 (13%)
Pain Management	3 (20%)
Pelvic Floor Physical Therapy	5 (33%)
Urology	1 (7%)
Integrative Medicine and Health	1 (7%)
Women’s Health Center	4 (27%)
Other	3 (20%)
None	3 (20%)
Pelvic examination findings	
Normal pelvic floor	2 (13%)
Pelvic exam not performed	1 (7%)
Trigger points and tenderness in levator ani muscles	12 (80%)
Trigger points and tenderness in obturator internus muscles	12 (80%)
Pudendal nerve tenderness	2 (13%)
Abdominal wall tenderness with positive Carnett sign	5 (33%)
Diffuse abdominal pain	3 (20%)
Adnexal tenderness	2 (13%)
Vulvovaginal atrophy or narrowed introitus	2 (13%)
Vaginal cuff tenderness	1 (7%)

During the study period, 14 patients attended the baseline individual mind-body counseling visit, 12 attended the first group visit, and nine attended the second group visit which demonstrates a 40% dropout rate by the completion of the mindfulness program. Despite decreased attendance in the latter sessions, all available PROMIS-CAT responses were included in the analysis. The median delay from enrollment to the first mind-body was 3.3 months (range = 1.1 to 4.7 months). The most common medical treatments during the six-month study period included initiation of pelvic floor physical therapy (N = 11) and prescription of vaginal diazepam (N = 8). Other treatments during the study period are described in Table 4.

**Table 4:** Treatments during the study period (N = 15).

	Median (minimum, maximum) or n (%)
Initiation of pelvic floor physical therapy	11 (73%)
Prescription of vaginal diazepam	8 (53%)
Pelvic floor trigger point injections	2 (13%)
Pelvic floor Botox injections	2 (13%)
Surgical intervention	5 (33%)
Other	2 (13%)
Initiation of hormone therapy for dysmenorrhea	2 (13%)
Prescription of vaginal estradiol cream	1 (7%)
Prescription of opioid medications	0 (0%)
Initiation of gonadotrophic-releasing hormone agonists or antagonists	1 (7%)
Total number of GYN clinic visits during the study period (telemedicine and in-person)	3 (1, 5)
Total number of patient-sent portal messages or phone calls during the study period	5 (0, 10)
Total number of ER visits (for any indication) during the study period	0 (0, 1)
Mind-body counseling visits attended	
Baseline individual visit	14 (93%)
First group visit	12 (80%)
Second group visit	9 (60%)
Time from enrollment to the first Mind-Body visit (months)	3.3 (1.1, 4.7)

**Figure 1:**
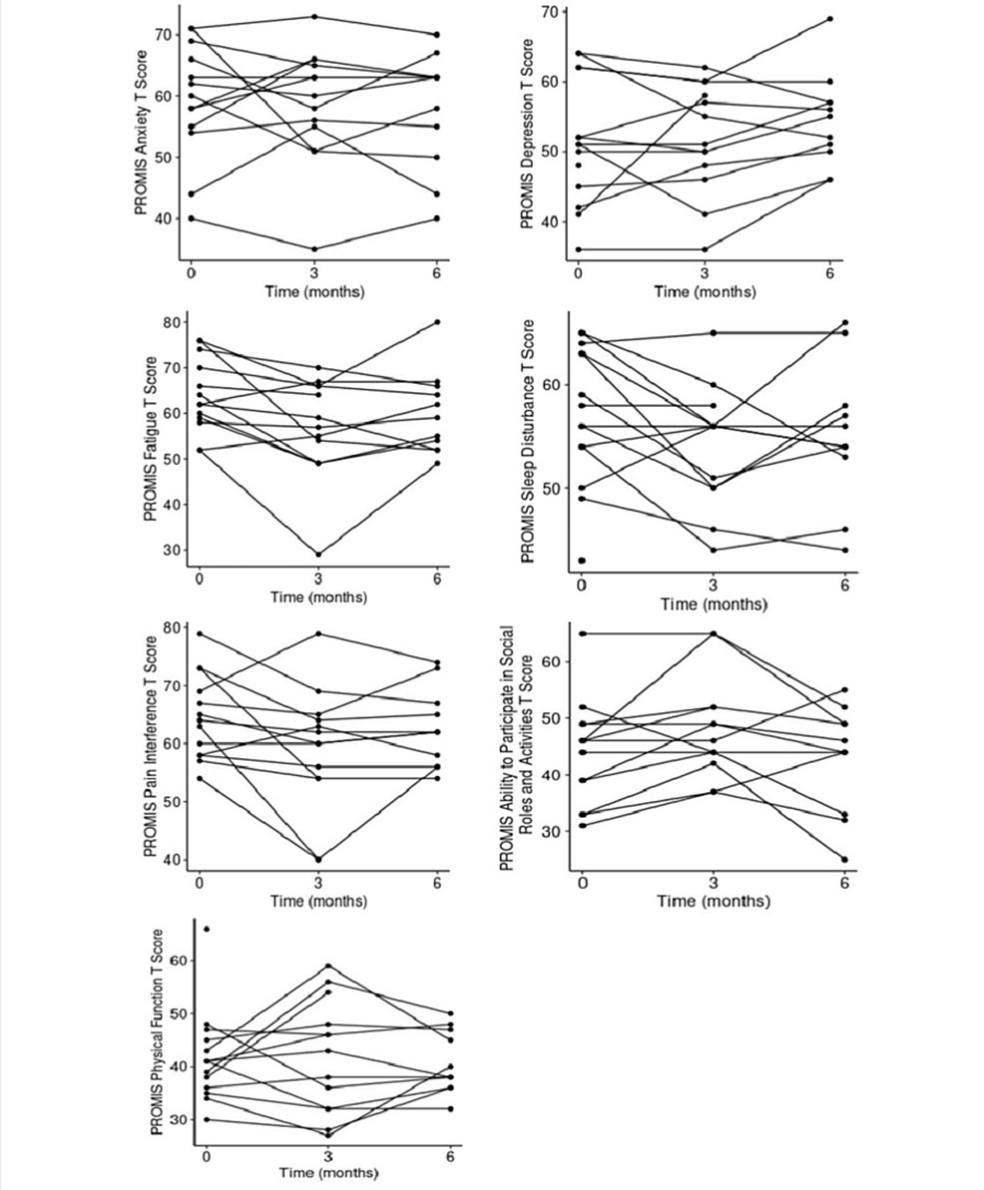
PROMIS-CAT T-score at baseline, three months, and six months. PROMIS-CAT = Patient-Reported Outcomes Measurement Information System-Computer Adaptive Testing

Table 5 describes the PROMIS-CAT scores at baseline, three months, and six months. Among the 13 patients who completed the three-month PROMIS-CAT scores, seven had at least a 5-point improvement in sleep disturbance T-score. At least a 5-point improvement in fatigue, pain interference, and ability to participate in social roles and activities T-scores were observed in six patients each (Table 6). Spaghetti plots for each of the seven PROMIS-CAT domains were compiled to show the trajectory of the scores over the study period (Figure 1).

**Table 5:** PROMIS-CAT scores at baseline and change from baseline to three and six months. Lower scores represent better outcomes for anxiety, depression, fatigue, sleep disturbance, and pain interference. Higher scores represent better outcomes for the ability to participate in social roles and physical function. PROMIS-CAT = Patient-Reported Outcomes Measurement Information System-Computer Adaptive Testing

PROMIS-CAT T-score	Baseline (N = 14)	Change from baseline to 3 months (N = 13)	Change from baseline to 6 months (N = 11)
Anxiety			
Mean (SD)	59 (9.232)	-0.692 (8.769)	-1.545 (7.133)
Median (Q1, Q3)	59 (55, 65)	0 (-5, 5)	0 (-2, 1)
Range	40 to 71	-20 to 11	-21 to 5
Depression			
Mean (SD)	51.429 (8.907)	0.154 (6.756)	1.818 (7.153)
Median (Q1, Q3)	51 (46, 60)	0 (-2, 1)	5 (-4, 7)
Range	36 to 64	-10 to 17	-12 to 10
Fatigue			
Mean (SD)	64.786 (8.487)	-7.462 (8.733)	-3.727 (9.089)
Median (Q1, Q3)	63 (59., 73)	-4 (-11, -2)	-4 (-7, 3)
Range	52 to 76	-23 to 5	-24 to 10
Sleep disturbance			
Mean (SD)	57.071 (6.696)	-4 (5.523)	-3.727 (5.120)
Median (Q1, Q3)	57 (54, 63)	-5 (-8, 0)	-5 (-7, 1)
Range	43 to 65	-13 to 6	-12 to 3
Pain interference			
Mean (SD)	64.571 (7.101)	-5.692 (9.205)	-2.818 (7.534)
Median (Q1, Q3)	64 (59, 69)	-3 (-10, -2)	-2 (-6, 2)
Range	54 to 79	-23 to 10	-19 to 6
Ability to participate in social roles			
Mean (SD)	44 (8.893)	4.154 (6.479)	0.182 (8.495)
Median (Q1, Q3)	45 (39, 48)	4 (0, 6)	3 (-7, 6)
Range	31 to 65	-8 to 19	-13 to 13
Physical function			
Mean (SD)	41.714 (8.624)	2.077 (9.465)	1.364 (6.516)
Median (Q1, Q3)	41 (37, 45)	2 (-3, 5)	2 (-1, 6)
Range	30 to 66	-12 to 17	-10 to 11

**Table 6:** PROMIS-CAT scores: percentage of patients who had at least a 5-point improvement from baseline to three months (N = 13). PROMIS-CAT = Patient-Reported Outcomes Measurement Information System-Computer Adaptive Testing

PROMIS-CAT T-score	n (%)
Anxiety	4 (31%)
Depression	2 (15%)
Fatigue	6 (46%)
Sleep disturbance	7 (54%)
Pain interference	6 (46%)
Ability to participate in social roles and activities	6 (46%)
Physical function	4 (31%)

## Discussion

This study demonstrated that the establishment of a mind-body counseling program that included individual and group components for a cohort of patients with chronic pelvic pain is feasible. Development of the program included the creation of the group virtual visit type, streamlining group scheduling, creation of a curriculum for the program, and collection of clinical data. Approximately 60% of participants attended all three sessions, and there were modest improvements in PROMIS-CAT domains over the study period. When utilizing an MCID of 5 points, there were improvements in T-scores across multiple domains in six of the 14 patients. The use of patient-reported outcomes measures is utilized in many areas of medicine, and the establishment of an MCID is helpful in the evaluation of the impact of an intervention on overall quality of life [14,16]. While our study did not validate the use of PROMIS-CAT in mind-body medicine for patients with pelvic pain, it aligns with the literature from other disciplines and can be used in clinical practice as a tool to monitor the effectiveness of a treatment course.

Qualitative feedback from the mindfulness sessions highlighted that being in a safe, positive community with other women as well as practicing holding and receiving compassionate awareness for and from others was deeply meaningful and impactful. Additional feedback from some of the participants included concerns that the program ended too quickly and more time working with the material was needed, as well as additional sessions with other group members to develop a sense of community. A systematic review by Jaderek et al. [5] found that studies utilizing mindfulness-based therapies for patients with sexual dysfunction are limited by variations in methodology, small participant sizes, patient selection, and variety of therapeutic interventions which limits their comparability. However, these studies have shown improvements in sexual arousal, desire, and satisfaction and reduction in anxiety following mindfulness therapy. Another systematic review by Samami et al. [17] on pain-focused psychological interventions in women with endometriosis showed that cognitive-behavioral therapy, mindfulness, yoga, psychoeducation, and progressive muscle relaxation training were all associated with a reduction in pain, as quantified by the Numeric Rating Scale, Endometriosis Health Profile, Visual Analog Scale, 26-item Short Form Health Survey, and the Chronic Pain Acceptance Questionnaire. Bittelbrunn et al. [18] also highlighted the importance of mindfulness and pelvic floor physical therapy as important components of pelvic pain management, shown by improvements in pain catastrophizing and sexual function after treatment with these interventions in their meta-analysis.

Strengths of this study include novel study design, completion of a small pilot trial within a 12-month time frame, and interdisciplinary collaboration between gynecology and integrative medicine and health practices. The mean waiting time from enrollment to the intervention was less than three months, which is reasonable given that there was only one counselor and schedule coordination for group sessions can be challenging.

Limitations include a small sample size as directed by the funding available for the project, lack of randomization or comparison groups, and loss of follow-up in 40% of subjects by the completion of the trial. While in-person counseling sessions were considered, we felt that access and attendance were improved with the virtual format, particularly due to the physical distancing constraints of the COVID-19 pandemic at the time of this study. It is possible that the pandemic affected the participant retention rate as there were many changes to healthcare and societal expectations during this time. Furthermore, there were limited times available for the group sessions, and lack of flexibility may have impacted the ability of patients to fully participate due to prior commitments and responsibilities. Additionally, patients had a variety of chronic pelvic pain diagnoses and treatments during the study period which resulted in significant heterogeneity of the group. This can cause biases and limit the generalizability of the results. Recruitment bias includes the exclusion of patients with a lack of access to electronic technology or the ability to attend virtual sessions due to other time commitments. Additionally, longer follow-up periods and additional assessment points could provide more comprehensive insights into the intervention’s sustained effects.

## Conclusions

Our goal from this initial trial is to explore the feasibility and efficacy of mind-body counseling in chronic pelvic pain. This study demonstrated a modest clinical improvement from enrollment in this program. We plan to design an adequately powered randomized controlled clinical trial with a treatment group that receives mind-body therapy and a control group that receives standard gynecologic care. Additional changes include limiting chronic pelvic pain diagnoses to dyspareunia and pelvic floor tension myalgia to have a more comparable cohort, including multiple mind-body counselors who develop and use a curriculum with reading assignments and activities that can provide better accountability and investment in the program, and use of a mixed-methods design using semi-structured interviews in addition to quantitative measures. We hope that additional attention to this topic will determine whether there is a role of integrative medicine and mind-body counseling in the management of chronic pelvic pain.
